# Protective effects of tasimelteon on kidney injury in a traumatic brain injury rat model: a histopathological and immunohistochemical study

**DOI:** 10.1007/s00068-025-02915-6

**Published:** 2025-06-27

**Authors:** Adem Milletsever, Halil Asci, Rumeysa Taner, Ozlem Ozmen

**Affiliations:** 1 Department of Pathology, Faculty of Veterinary Medicine, Burdur Mehmet Akif Ersoy University, Burdur, Türkiye; 2 Department of Pharmacology, Faculty of Medicine, Suleyman Demirel University, Isparta, Türkiye; 3 Department of Bioengineering, Institute of Natural and Applied Sciences, Suleyman Demirel University, 32200 Isparta, Türkiye

**Keywords:** Brain, Kidney, Pathology, Tasimelteon, Trauma

## Abstract

**Purpose:**

Traumatic brain injury (TBI) is a condition characterized by structural and functional damage to the brain following trauma and is a significant cause of mortality. Acute kidney injury (AKI) has been reported in patients with TBI and is a commonly encountered complication. This study examines the effect of tasimelteon (Tasi) application on kidney tissue in TBI rats by histopathological and immunohistochemical staining.

**Methods:**

Forty male Wistar Albino rats weighing 300–350 g were randomly divided into four groups: Control group, Trauma group (Trau), Trau-Tasi-1 group (trauma-1 mg/kg Tasi intraperitoneally), and Trau-Tasi-10 group (trauma-10 mg/kg Tasi intraperitoneally). At the end of the experimental phase, after euthanasia, kidney tissue was collected for histopathological and immunohistochemical analyses.

**Results:**

The results of histopathological and immunohistochemical staining demonstrate that brain trauma may induce kidney injury, while Tasi possesses the potential to ameliorate kidney lesions associated with TBI. It has been found that Trau-Tasi-10 is more effective in treatment compared to Trau-Tasi-1.

**Conclusion:**

We observed Tasi’s kidney injury ameliorating activity in TBI rats through histopathological and immunohistochemical staining findings.

**Supplementary Information:**

The online version contains supplementary material available at 10.1007/s00068-025-02915-6.

## Introduction

Traumatic brain injury (TBI) is a condition characterized by structural or functional damage to the brain resulting from a traumatic event, and it is a significant cause of hospitalizations and mortality. Nearly half of patients with TBI are admitted to intensive care units, where systemic disease is frequently observed. Additionally, acute kidney injury (AKI) is reported in TBI patients at variable incidences and is a common complication. Consequently, many patients with TBI are at risk of developing AKI, and there is an association between increased mortality and the development of AKI following TBI [1, 2].

The pathophysiology of AKI in TBI is not fully understood; however, systemic inflammation through molecules such as interleukins and cytokines released into circulation due to tissue damage, along with autonomic dysfunction, plays a significant role. TBI leads to an inflammatory response marked by the release of cytokines in the cerebrospinal fluid and serum. Interleukin-6 (IL-6) has been associated with AKI [1, 2]. In animal models of TBI, elevated expression levels of interleukins, such as IL-6, are observed following injury [3]. IL-6 and inflammatory mediators cause renal cell damage. Additionally, immediately after TBI, IL-6, IL-8, and IL-10 are strongly associated with dysfunction in renal and other organ systems, as well as with multiple organ dysfunction syndrome. Furthermore, increased neutrophil adhesion and apoptosis in renal tubular cells have been observed in TBI patients [4, 5].

Another pathway through which renal dysfunction can be induced in TBI involves catecholamines. Catecholamines activate the renin-angiotensin-aldosterone system, affecting renal function by impacting renal perfusion through vasoconstriction of afferent renal arteries and increased sodium reabsorption [6]. Therapeutic strategies aimed at preventing secondary injury after TBI may also influence renal function. Additionally, AKI can impact brain function by increasing blood-brain barrier permeability and cerebral inflammation. Current treatment options for the prevention and management of AKI following TBI remain limited [7, 8].

Melatonin (MEL) is a pleiotropic neurohormone primarily produced in the pineal gland. While various tissues secrete it, it also acts as a local regulatory molecule [9]. The primary role of MEL is to convey information related to the daily light-dark cycle to different regions of the human body, which ultimately affects the functioning of the entire organism [10]. However, numerous reports indicate that this is not this molecule’s only mechanism and function. MEL has also been shown to participate in antioxidant, anti-inflammatory, anti-apoptotic, and immune processes [10–15]. The renoprotective effect of MEL has been the subject of reports over the past decade, revealing that MEL not only improves sleep disturbances in patients with chronic kidney disease (CKD) but also has beneficial effects on blood pressure. Additionally, it provides protection against oxidative stress and inflammation observed in various kidney injuries, such as CKD, glomerulonephritis, contrast-induced kidney injury, drug-induced nephrotoxicity, and acute ischemia-reperfusion injury [16–18]. This is consistent with the definition of AKI in the Kidney Disease: Improving Global Outcomes (KDIGO) guidelines. This condition results from the direct effect of contrast agents on the kidneys, manifesting as damage to the tubular epithelial cells. Furthermore, vasoactive molecules are released, leading to ischemic injury by inducing oxidative stress [19].

Tasimelteon (Tasi) is a selective MEL receptor agonist primarily approved for the treatment of non-24-hour sleep-wake disorder, owing to its chronobiotic properties. Notably, it exhibits a higher binding affinity for MEL receptor 2 (MT2) compared to MEL receptor 1 (MT1) [20]. Beyond its role in regulating circadian rhythms, growing evidence suggests that activation of MEL receptors plays a pivotal role in modulating immune responses, exerting anti-inflammatory, anti-apoptotic, and antioxidant effects. Furthermore, MEL receptor activation has been associated with protective effects against ischemia/reperfusion (I/R) injury in various tissues, highlighting their potential as promising therapeutic targets for organ protection [21, 22].

In this context, Tasi may offer cytoprotective benefits by preserving mitochondrial function, reducing oxidative stress, inhibiting apoptotic pathways, and suppressing pro-inflammatory cytokine expression [22]. Based on these mechanisms, the present study was conducted to investigate the potential protective effects of Tasi on AKI, particularly in the setting of TBI. For this purpose, histopathological and immunohistochemical methods were employed to evaluate renal tissue damage and the therapeutic potential of Tasi.

## Materials and methods

### Animals and ethical approval

The material for this study was obtained from the kidneys of rats previously used in our team’s TBI research. Ethical approval was obtained to utilize the kidney tissues for the purposes of this study [22].

Forty adult male Wistar albino rats weighing 300–350 g were housed in standard Euro-type 4 cages, each group separated from the other. They were housed at 23 °C and 55% humidity with 12 h of light and 12 h of dark cycle. They were fed ad libitum with a standard commercial feed and water. The four experimental groups were formed as follows:

- Control group (*n* = 10): Rats were administered 0.5–1 ml saline (SF) intraperitoneally (IP) without trauma. After 24 h, rats were euthanized under anesthesia, and kidney tissues were collected.
- Trauma group (*n* = 10): Rats were administered 0.5–1 ml SF IP 30 min under anesthesia after trauma was induced. After 24 h, rats were euthanized under anesthesia, and kidney tissues were collected.
- Trau-Tasi-1 group (*n* = 10): Rats were administered 1 mg/kg Tasi (SML2030, Sigma Aldrich, USA) Tasimelteon IP under anesthesia 30 min after trauma was induced [23]. After 24 h, rats were euthanized under anesthesia, and kidney tissues were collected.
- Trau-Tasi-10 group (*n* = 10): Rats were traumatized under anesthesia. After 30 min, 10 mg/kg Tasi was administered IP [23]. After 24 h, rats were euthanized under anesthesia, and kidney tissues were collected.

**Fig. 1:**
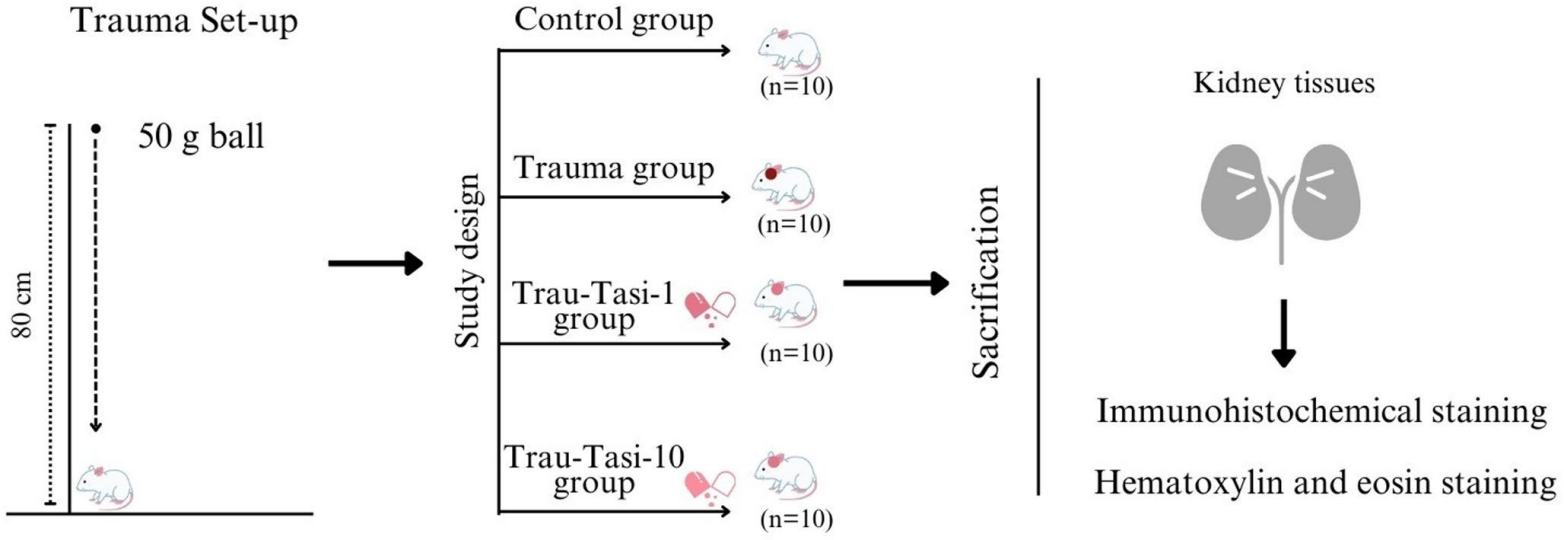
Study Design and Trauma Set-up. Trau-Tasi-1 group: Trauma-Tasimelteon 1 mg/kg group; Trau-Tasi-10 group: Trauma-Tasimelteon 10 mg/kg group

Head trauma was induced using a 50 g ball dropped from a height of 80 cm based on the shock acceleration model of Cıkrıklar et al. [24] Fig. 1.

Rats were anesthetized with an intraperitoneal injection of ketamine (90 mg/kg) (Keta-Control, Doğa İlaç, Türkiye) and xylazine (10 mg/kg) (Xylazin Bio 2%, Bioveta, Czech Republic) prior to the induction of TBI and before sacrifice. Rats were anesthetized with The TBI was induced using the impact acceleration model described previously. Throughout the procedure, animals were monitored for signs of adequate anesthesia and maintained under anesthesia until the injury was completed. After TBI induction, rats were allowed to recover under standard laboratory conditions until sacrifice at the predetermined time points. For the euthanasia procedure, surgical exsanguination was performed by drawing blood from the inferior vena cava. After euthanasia, kidney tissue was collected. Kidney tissues were placed in formaldehyde solution for histopathological examination. Kidney tissues were stained with hematoxylin and eosin (HE) for histopathology. The levels of pathological findings such as hemorrhage, inflammation, and necrosis were examined by HE staining, and haptoglobin, malondialdehyde, and transient receptor potential cation channel subfamily M member 2 (TRPM-2) were analyzed by immunohistochemical staining.

### Histopathological method

Kidney samples were collected during necropsy and subjected to a macroscopic examination for any pathological findings before being fixed in a 10% neutral formalin solution. The tissue samples were processed using the Leica ASP300S automatic tissue processing equipment and subsequently embedded in paraffin wax. After cooling, 5 μm thick sections were obtained from the paraffin blocks with a Leica RM2155 rotary microtome. These sections were then stained with hematoxylin-eosin (HE) and examined under a light microscope.

The histological lesions in the tissues were assessed using an ordinal grading system that evaluated hyperemia, hemorrhage, inflammatory cell infiltrations, degenerative changes, and necrotic changes. Normal tissues were assigned a score of 0, while mild, moderate, and severe affections were scored as 1, 2, and 3, respectively. The criteria for scoring the histopathological changes are detailed in Table 1.

**Table 1:** Histopathological grading scores

Score	Description
0	No lesion
1	Mild hyperemia, slight hemorrhage, no inflammation and vacuolar degeneration
2	Severe hyperemia, slight hemorrhage, slight inflammation and slight necrosis
3	Severe hyperemia, severe hemorrhage, marked inflammation and marked necrosis

### Immunohistochemical examination

Additionally, three sets of sections were prepared from all blocks on poly-L-lysine-coated slides and underwent immunohistochemical staining for HP (haptoglobin) (Anti-Haptoglobin antibody [EPR22856-212] (ab256454)), MDA (malondialdehyde) (Anti-Malondialdehyde antibody [11E3] (ab243066)), and Transient Receptor Potential Melastatin 2 (TRPM-2) (Anti-TRPM-2 antibody (ab11168) (Abcam, Cambridge, UK)) using the streptavidin-biotin technique, following the manufacturer’s instructions. The primary antibodies were diluted at a ratio of 1:100 and incubated with the sections for 60 min. This was followed by immunohistochemistry using biotinylated secondary antibodies and streptavidin-alkaline phosphatase conjugate. The EXPOSE Mouse and Rabbit Specific HRP/DAB Detection IHC kit (ab80436) (Abcam, Cambridge, UK) served as the secondary antibody, while diaminobenzidine (DAB) was used as the chromogen. Negative controls were treated with an antigen dilution solution instead of primary antibodies. Each test was conducted by a trained pathologist from another university who was blinded to the samples.

Immunohistochemical analysis was performed separately for each antibody. All evaluations were conducted in a blinded manner. At a magnification of ×40, 100 cells were counted from each group to determine the percentage of positive cells for each marker in each area. The ImageJ program (version 1.48, National Institutes of Health, Bethesda, MD) was utilized to analyze the images. Statistical analysis was performed on the data generated by the image analyzer. Morphometric studies were conducted using the Database Manual CellSens Life Science Imaging Software (Olympus Co., Tokyo, Japan). The ImageJ 1.48 program (National Institutes of Health, Bethesda, MD) was also used to evaluate immunopositive cell scores.

### Statistical analysis

Shapiro-Wilk method was employed to assess the normality of the data distribution. ANOVA was employed as a means of comparing the groups since the data showed a normal distribution (*P* > 0.05). The results were analyzed for normality distribution, and one-way ANOVA followed by Tukey’s multiple comparison test was used. Statistical analysis was performed using GraphPad Prism 8.0 (GraphPad Software, MA, USA). Differences were considered significant for *p* < 0.05. All results are expressed as mean ± SD Table 2.

## Results

### Tasi significantly decreased the pathological findings related to TBI-induced AKI

**Fig. 2:**
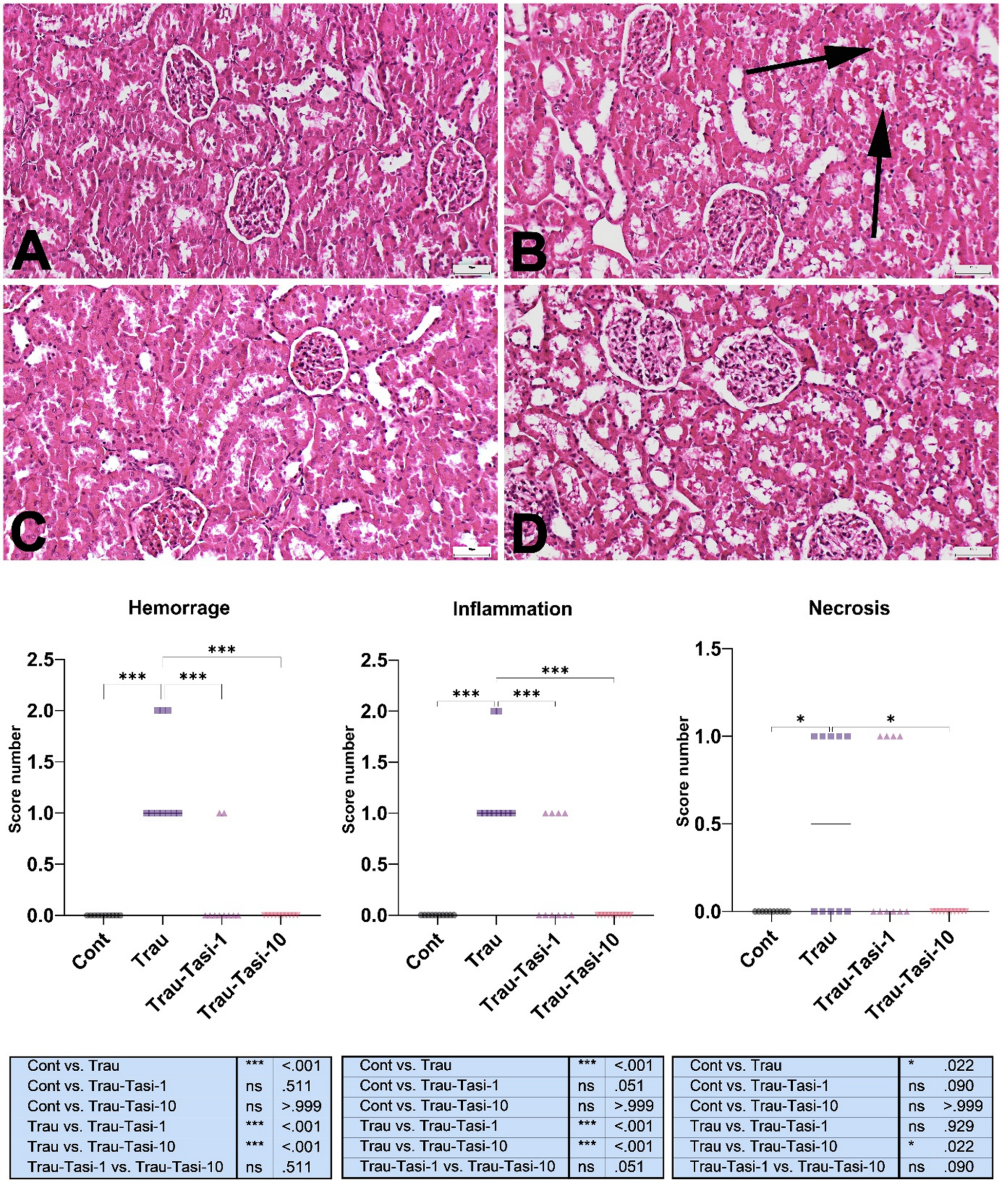
Histopathological appearance of the kidney between the groups. (A) Normal tissue histology in the Control group. (B) Proteinous fluid in the tubular lumens (arrows) in the Trau group. (C) Decreased pathological findings in the Trau-Tasi-1 group. (D) Marked amelioration in the Trau-Tasi-10 group. HE, Scale Bars = 50 μm. Graphs display the statistical analysis of histopathological scores. Values are presented as the mean ± SD. Abbreviations: Cont: Control, Trau: Trauma, Tas-1: Tasimelteon 1 mg/kg, Tasi-10: Tasimelteon 10 mg/kg, ***:*p* < 0.001 *:*p* < 0.05

Histopathological examination revealed normal kidney morphology in the Control group. In contrast, the Trau group showed marked hyperemia, microhemorrhages, and varying degrees of inflammatory cell infiltration. Additionally, some rats exhibited proteinaceous material within the tubular lumen and glomeruli. Evaluation of hemorrhage and inflammation demonstrated significant differences between the Control and Trauma groups (*p* < 0.001) as well as between the trauma and treatment groups (*p* < 0.001). However, no significant differences were observed between the treatment groups themselves (*p* > 0.05) or between the treatment groups and the Control group (*p* > 0.05). Regarding necrosis, the Trauma group exhibited a significant increase compared to controls (*p* = 0.022), whereas treatment with Tasi at 10 mg/kg (Trau-Tasi-10 group) effectively alleviated pathological alterations, with the higher dose producing a more pronounced improvement (Fig. 2).

### Tasi’s beneficial efficacy on AKI post-TBI is dose-dependent

**Fig. 3:**
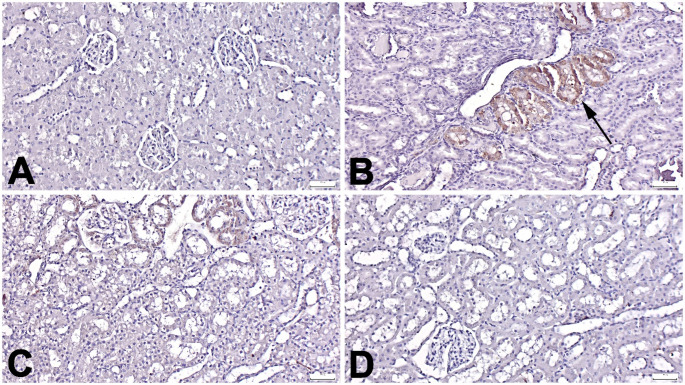
Representative haptoglobin immunohistochemical findings among the groups. (A) Negative expressions in the Control group. (B) Marked increased expressions (arrows) in the Trau group. (C) Markedly decreased expressions in the Trau-Tasi-1 group. (D) No expression in the Trau-Tasi-10 group. Streptavidin Biotin Peroxidase Method, Scale Bars = 50 μm. Trau: Trauma, Trau-Tas-1: Tasimelteon 1 mg/kg, Trau-Tasi-10: Tasimelteon 10 mg/kg

**Fig. 4:**
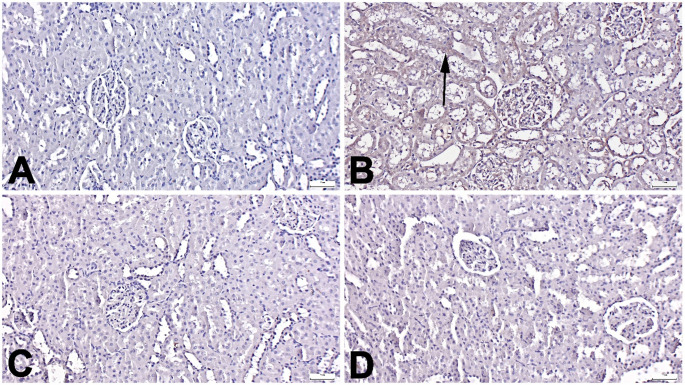
Representative malondialdehyde immunohistochemical findings among the groups. (A) Negative expressions in the Control group. (B) Marked increased expressions (arrows) in the Trau group. (C) Markedly decreased expressions in the Trau-Tasi-1 group. (D) No expression in the Trau-Tasi-10 group. Streptavidin Biotin Peroxidase Method, Scale Bars = 50 μm. Trau: Trauma, Tas-1: Tasimelteon 1 mg/kg, Tasi-10: Tasimelteon 10 mg/kg

**Fig. 5:**
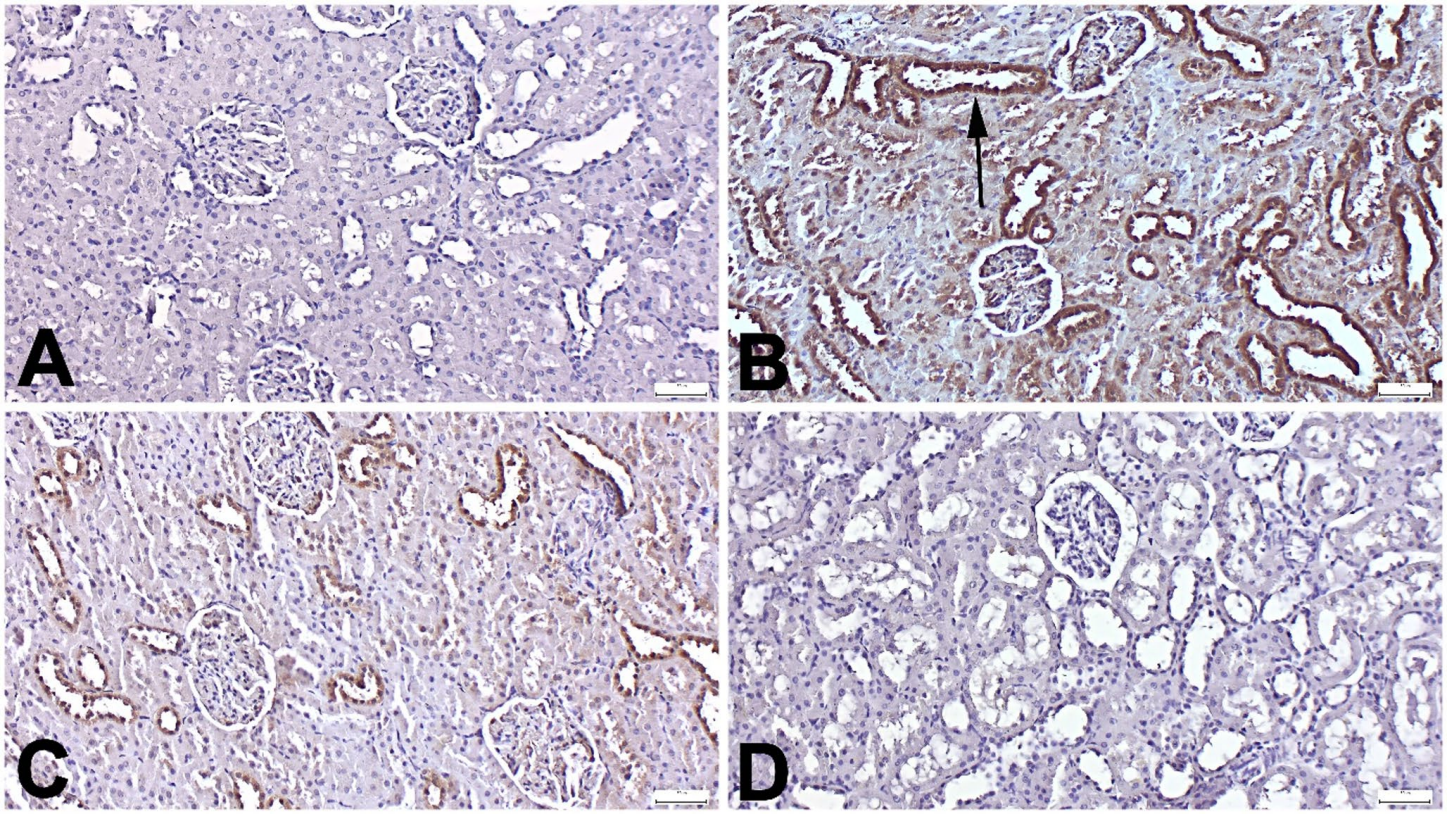
Representative TRPM-2 immunohistochemical findings among the groups. (A) Negative expressions in the Control group. (B) Marked increased expressions (arrows) in the Trau group. (C) Markedly decreased expressions in the Trau-Tasi-1 group. (D) No expression in the Trau-Tasi-10 group. Streptavidin Biotin Peroxidase Method. Scale Bars = 50 μm. TRPM-2: Transient receptor potential cation channel subfamily M member 2, Trau: Trauma, Trau-Tas-1: Tasimelteon 1 mg/kg, Trau-Tasi-10: Tasimelteon 10 mg/kg

**Fig. 6:**
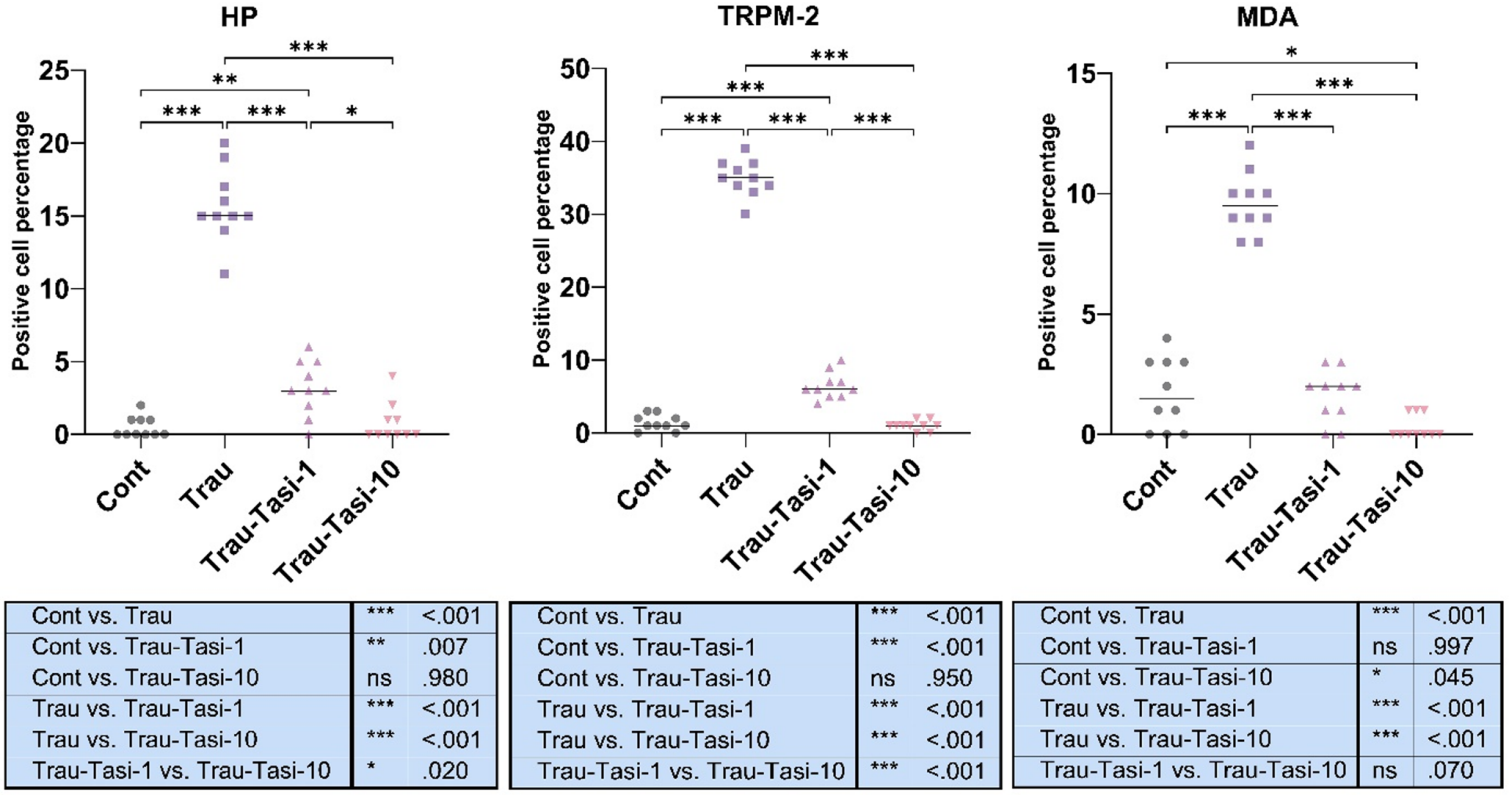
Statistical analysis of immunohistochemical expressions. Graphs display the statistical analysis of Cas-3 immunoscores. Data presented as the mean ± SD. Abbreviations: Cont: Control; Trau: Trauma; Trau-Tas-1: Tasimelteon 1 mg/kg; Trau-Tasi-10: Tasimelteon 10 mg/kg. *:*p* < 0.05, **p < 0.01, ***:*p* < 0.001

Microscopical examination of HP, MDA, and TRPM-2 immunostained slides showed no or very few immunopositive cells in the Control group. In contrast, a marked increase in expression was observed in the Trau group. Positive reactions were noted in endothelial cells, tubular epithelial cells, and interstitial cells. However, Tasi treatment significantly reduced these expressions, with Trau-Tasi-10 exhibiting a more pronounced ameliorative effect than Trau-Tasi-1 (*p* < 0.001 for all). A significant difference in HP and TRPM-2 expression was observed between the Trau-Tasi-10 and Trau-Tasi-1 groups (*p* < 0.05 and *p* < 0.001, respectively) (Figs. 3, 4, 5 and 6). The results of the study indicate that trauma can lead to kidney damage, whereas Tasi has the potential to ameliorate TBI induced kidney lesions. Tasi-10 was found to be more effective than Tasi-1 in treatment. Thus, the effect of tasi on kidney damage varies depending on the dose.

**Table 2:** Immunohistochemical positivity (%) for haptoglobin (HP), malondialdehyde (MDA), and TRPM-2 in kidney tissues across groups

Group	HP (%)	MDA (%)	TRPM-2 (%)
Control	0.50 ± 0.70	1.70 ± 1.49	1.40 ± 1.07
Trau	15.70 ± 2.54	9.60 ± 1.26	35.00 ± 2.49
Trau-Tasi-1	3.20 ± 1.87	1.60 ± 1.07	6.50 ± 1.84
Trau-Tasi-10	0.80 ± 1.31	0.30 ± 048	1.00 ± 0.66

Data are presented as mean ± SD. Trau: Trauma, Trau-Tas-1: Tasimelteon 1 mg/kg, Trau-Tasi-10: Tasimelteon 10 mg/kg

## Discussion

In this study, we firstly investigated the effect of Tasi, a MEL agonist, on kidney damage observed after TBI using histopathological and immunohistochemical methods. Recent studies have reported that the incidence of AKI in patients with severe TBI ranges from 8 to 23%, particularly in those requiring intensive care support. For instance, Huang et al. (2022) and Ramírez-Guerrero et al. (2022) emphasized that AKI represents a significant extracranial complication in severe TBI patients, often associated with increased morbidity and mortality [25, 26]. Similarly, Corral et al. (2012) highlighted that non-neurological complications, including renal injury, can substantially worsen the prognosis of TBI patients [27]. Although mild and moderate TBI are not typically linked to renal dysfunction, severe TBI triggers systemic inflammatory responses, sympathetic hyperactivation, and oxidative stress, all of which can contribute to secondary organ injuries, including the kidneys [28–30]. Based on this evidence, our study aimed to investigate the histopathological and immunohistochemical alterations in renal tissue following TBI and to evaluate the potential protective effects of Tasi well-documented antioxidant and anti-inflammatory properties. Histopathologically, Tasi treatment significantly reduced renal lesions and inflammation. Additionally, Tasi treatment demonstrated a therapeutic effect on the kidney by significantly reducing the expression of HP, MDA, and TRPM-2 in immunohistochemical analyses. The brain and kidneys are interrelated, with their connection mediated by vasoregulatory systems and humoral pathways. Therefore, any change in one organ can influence the other [28]. Following TBI, extracranial organ dysfunction, including renal system impairment, is observed. While direct trauma leads to primary brain injury, damage to extracranial organs occurs through autonomic and inflammatory pathways in response to the initial insult to the brain [1, 2]. TBI induces an inflammatory response through the autonomic nervous system and cytokines released from the brain. Neurons within various brain nuclei regulate renal hemodynamics; thus, damage to the brain parenchyma results in increased sympathetic activity [31, 32]. This hyperactive state leads to renal vasoconstriction, reduced renal perfusion, and decreased glomerular filtration rate.

Additionally, increased renal clearance is observed post-TBI [33]. Consequently, alterations in renal perfusion are noted following brain injury. Sympathetic stimulation causes elevated glucocorticoid levels and concurrent activation of the renin-angiotensin-aldosterone system, playing a critical role in the pathogenesis of kidney damage. Angiotensin II activates the expression of inflammatory factors, such as cytokines, IL-6, and reactive oxygen species (ROS), in the kidney, inducing inflammatory cell infiltration in glomerular and tubular cells [34, 35]. Cellular damage resulting from TBI triggers the release of molecular substances that promote the release of inflammatory mediators and the infiltration of immune cells into the brain [31]. Moreover, the breakdown of the blood-brain barrier following TBI facilitates the entry of cytokines into the systemic circulation, rendering the kidney vulnerable to dysfunction through this systemic inflammatory response [33].

Functional alterations and apoptosis are observed in tubular epithelial cells post-TBI. Studies have demonstrated that various types of renal injury trigger an acute inflammatory response along with cytokine release and immune system activation [36]. In this study, prominent hyperemia, microhemorrhage, and inflammatory cell infiltration observed histopathologically in the trau group support the inflammatory response and cellular damage associated with AKI following TBI. The Trau-Tasi-1 and Trau-Tasi-10 groups reduced these inflammatory responses and improved histopathological lesions. It is suggested that Tasi may have a protective effect on the kidney; however, this protective effect should be further supported by additional studies and molecular methods.

In AKI observed following TBI, elevated levels of ROS due to cytokines and oxidative stress cause damage to glomerular and tubular cells. Additionally, inflammatory cell infiltrations are observed in AKI [37]. MDA, a marker of oxidative stress, shows increased expression during lipid peroxidation. Oxidative stress, driven by elevated ROS levels, leads to cellular damage [30]. Another protein that plays a crucial role in oxidative cellular damage is TRPM-2. TRPM-2 senses oxidative stress, becomes activated, and contributes to oxidative stress. Additionally, TRPM-2 serves as a critical mediator in renal epithelial cell apoptosis and neutrophil infiltration [38, 39]. Another protein that shows increased expression due to inflammation and oxidative stress is haptoglobin (HP) [39]. Significant increases in HP expression are observed in various AKI models, reflecting tissue damage responses [40]. In this study, the immunohistochemical increase in the expression of oxidative stress markers HP, MDA, and TRPM-2 in the trauma group indicates the development of trauma-induced oxidative stress. Tasi treatment significantly reduced these expressions, with Trau-Tasi-10 demonstrating a more pronounced therapeutic effect than Trau-Tasi-1. This suggests that Tasi may have antioxidant properties. Additionally, the parallel reduction in inflammation and oxidative stress with Tasi treatment further supports this possibility. However, to confirm Tasi’s antioxidant properties, further analysis of antioxidant markers with Tasi alone is necessary.

This study is the first to investigate the pathological effects of Tasi on AKI following TBI. Our findings provide novel insights into the potential renoprotective properties of Tasi in this context.

This study has several limitations. Firstly, the protective effects of Tasi on the kidney were evaluated using histopathological and immunohistochemical methods, but the underlying molecular mechanisms were not investigated. Therefore, it is important for future studies to verify the antioxidant and anti-inflammatory effects of Tasi through molecular biology techniques. Additionally, the study was limited to only two doses of Tasi, and different dosage ranges or treatment durations were not assessed; thus, further research is needed to clarify the dose-response relationship and determine the optimal treatment regimen. Functional kidney parameters, such as serum creatinine and blood urea nitrogen (BUN), were not measured, which precluded correlating histological findings with functional outcomes. Moreover, changes in kidney injury over time following TBI and the long-term effects of Tasi were not explored in this study.

## Conclusion

In conclusion, this study suggested that Tasimelteon, a melatonin agonist, may provide protection against acute kidney injury caused by traumatic brain injury by decreasing histopathological lesions and inflammation. Tasimelteon treatment significantly decreased the immunohistochemistry expression of oxidative stress markers such haptoglobin, malondialdehyde, and TRPM-2, indicating that it may have anti-oxidative effects. The reduction of inflammation and oxidative stress by Tasimelteon administration may have a therapeutic potential in preventing acute kidney injury associated with traumatic brain injury. In addition, the more pronounced protective effects observed in the Trau-Tasi-10 group compared to Trau-Tasi-1 suggested that Tasi may have a dose-dependent efficacy in reducing kidney injury. Further studies are required to confirm the antioxidant capacity of Tasi and its potential clinical applications.

## Electronic supplementary material

Below is the link to the electronic supplementary material.

Supplementary material 1 (JPG 973 KB)

## Data Availability

No datasets were generated or analysed during the current study.
