# Supplementary figures and images for: Protective effects of tasimelteon on kidney injury in a traumatic brain injury rat model: a histopathological and immunohistochemical study
Source: Eur J Trauma Emerg Surg. 2025 Jun 27;51(1):241. doi: 10.1007/s00068-025-02915-6 (PMC12205017; doi:10.1007/s00068-025-02915-6)

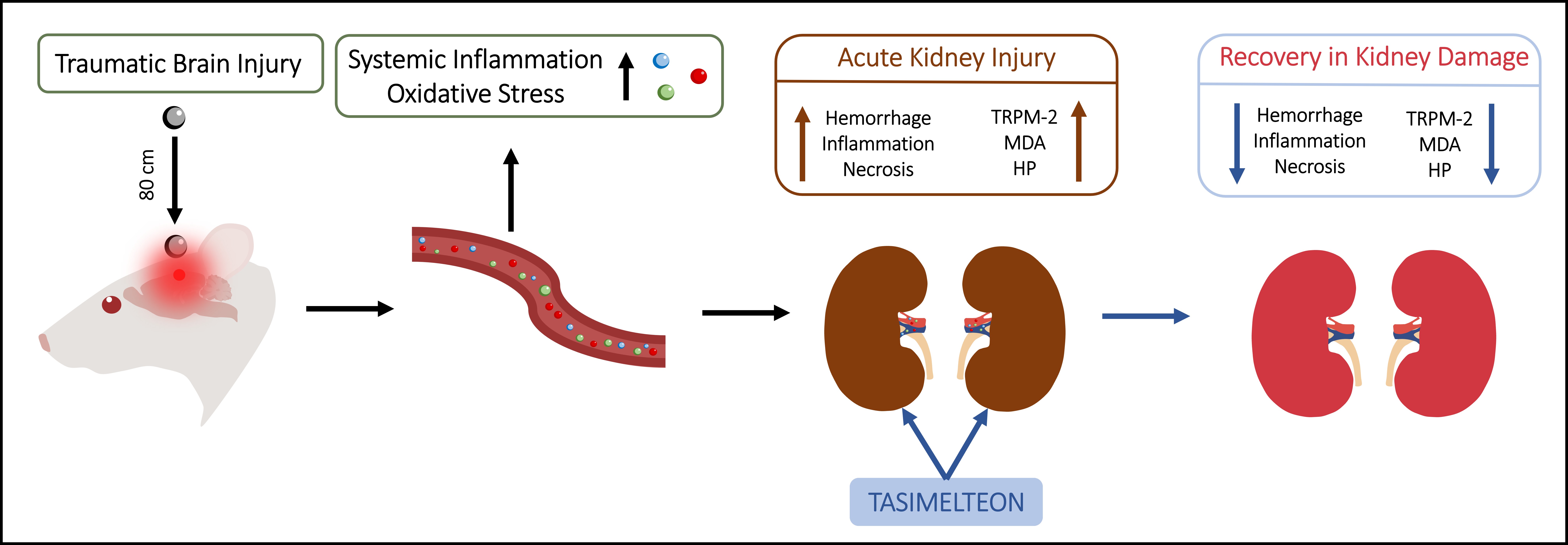

Supplement: Supplementary file 1 — Supplementary material 1 (JPG 973 KB) [file 68_2025_2915_MOESM1_ESM.jpg]
